# Adult attention deficit hyperactivity disorder is associated with asthma

**DOI:** 10.1186/1471-244X-11-128

**Published:** 2011-08-07

**Authors:** Ole Bernt Fasmer, Anne Halmøy, Tomas Mikal Eagan, Ketil Joachim Oedegaard, Jan Haavik

**Affiliations:** 1 Department of Clinical Medicine, Section for Psychiatry, University of Bergen, Bergen, Norway; 2 Division of Psychiatry, Haukeland University Hospital, Bergen, Norway; 3 K.G. Jebsen Centre for Research on Neuropsychiatric Disorders, Bergen, Norway; 4 Department of Biomedicine, University of Bergen, Bergen, Norway; 5 Department of Thoracic Medicine, Haukeland University Hospital, Bergen, Norway; 6 Institute of Medicine, Section for Thoracic Medicine, University of Bergen, Bergen, Norway

## Abstract

**Background:**

Attention deficit hyperactivity disorder (ADHD) is increasingly recognized as a common disorder not only in children, but also in the adult population. Similarly, asthma also has a substantial prevalence among adults. Previous studies concerning a potential relationship between ADHD and asthma have not presented consistent results.

**Methods:**

A cross-sectional study of 594 adult patients diagnosed with ADHD, compared with 719 persons from the general population. Information was collected between 1997 and 2005 using auto-questionnaires rating past and present symptoms of ADHD, co-morbid conditions, including asthma, and work status.

**Results:**

The prevalence of asthma was significantly higher in the ADHD patient group compared to the controls, 24.4% vs. 11.3% respectively (OR = 2.54, 95% CI 1.89-3.44), and controls with asthma scored higher on ratings of both past and present symptoms of ADHD. Female ADHD patients had a significantly higher prevalence of asthma compared to male ADHD patients (30.9% vs. 18.2%, OR = 2.01, CI 1.36-2.95), but in controls a slight female preponderance was not statistically significant. In both ADHD patients and controls, having asthma was associated with an increased prevalence of symptoms of mood- and anxiety disorders.

**Conclusions:**

The present findings point to a co-morbidity of ADHD and asthma, and these patients may represent a clinical and biological subgroup of adult patients with ADHD.

## Background

Attention deficit hyperactivity disorder (ADHD) is a common disorder in children causing substantial problems for those afflicted [1-3]. During the last 10-15 years ADHD has been the focus of increasing interest also in adult psychiatry [2-4]. The prevalence of ADHD is estimated to be in the range of 2-12% in children [5-7] and 3-5% in adults [8,9]. Many children with ADHD retain impairing symptoms as adults, causing difficulties in relation to educational, social and occupational functioning [10,11]. In addition, ADHD is associated with many other psychiatric disorders, in particular anxiety- and mood disorders [9,11].

Like ADHD asthma is also perceived as a disease of childhood, however with a significant adult prevalence and incidence [12-14]. Both asthma [15] and ADHD [16] have a clear genetic component. Asthma has a well established co-morbid connection with psychiatric disorders. Children with asthma have a higher prevalence of behavioural difficulties than children without asthma [17]. Among adult asthmatics, there is a higher prevalence of depression and anxiety disorders than in the general population [18-20].

The pathophysiology and genetics of ADHD probably involve multiple neurotransmitter systems, including dopaminergic mechanisms [21], but a comprehensive understanding of this disorder is still lacking. Pelsser, Buitelaar & Savelkoul [22] have advanced the hypothesis that ADHD may be a non-allergic hypersensitivity disorder, with pathophysiological links to asthma.C

Previous studies that have examined the potential relationship between ADHD and asthma have not presented consistent results. Both a study of 140 boys with ADHD [23] and a study of 140 girls with ADHD [24] failed to find a positive association between ADHD and asthma. However, in a large study from the National Survey of Children's Health in the USA, children with asthma were more likely to have co-morbid ADHD compared with children without asthma [25]. A previous study on adults, with data from a large claims database in the USA, showed that ADHD was significantly associated with asthma [26].

In a study using data from the Norwegian Prescription Database we have shown that patients prescribed drugs to treat ADHD also are prescribed anti-asthma drugs significantly more often than the rest of the population [27].

The aims of the present study were to (1) investigate the prevalence of asthma among clinically diagnosed adults with ADHD, compared to controls from a normal population, and to (2) investigate if the presence of asthma is associated with differences in symptom patterns and demographic variables in patients and controls.

## Methods

### Subjects

This is a cross-sectional study of 594 Norwegian patients diagnosed with adult ADHD and a comparison group of 719 persons from the general population. The patients were recruited as part of a genetic study using a national registry of adults diagnosed with ADHD in Norway during 1997-May 2005 [11,21,28,29]. The diagnostic assessment of the patients in the registry was made by one of three national expert committees for ADHD, and was based on detailed clinical information (including information from informants) provided by the referring clinicians, mainly psychiatrists. The diagnosis of ADHD was made according to the ICD-10 research criteria, with two modifications; allowing the inattentive subtype, as in DSM-IV, as sufficient for the diagnosis, and allowing for the presence of co-morbid psychiatric disorders, as long as the criteria for ADHD were present before the appearance of the co-morbid disorder.

To enhance recruitment and to include patients diagnosed also later than May 2005, psychiatrists and psychologists nation-wide were invited to recruit formally diagnosed adult patients with ADHD. The inclusion criteria were a diagnosis of ADHD according to the criteria described above, and age above 18 years. There were no formal exclusion criteria. In the present paper we report on data from a total of 594 patients, 340 from the national registry, and 254 from the recruitment performed after May 2005.

The control group was recruited using the database of The Medical Birth Registry of Norway (MBRN). The MBRN includes all people born in Norway after January 1st 1967. Invitation letters were sent out in 2007-2009 to a randomly selected sample of persons between 18-40 years from all over Norway. Data from the first 719 persons recruited are presented in the present report. For further details about the recruitment strategy and the patient sample, see Johansson et al. 2008 [21], Halleland et al. 2009 [28], Halmøy et al. 2009 [11], and Halmøy et al. 2010 [29].

Informed consent based on detailed written information about the project was obtained from all patients and controls. The study was approved by the Regional Research Ethical Committee of Western Norway.

### Questionnaires

The following self-report questionnaires were used in this study: The Wender Utah Rating Scale (WURS), measuring the presence and frequency of childhood ADHD symptoms [30], the Adult ADHD Self Report Scale (ASRS) which measures the presence and frequency of current symptoms of ADHD [31,32], and the Mood Disorder Questionnaire (MDQ), a screening questionnaire for bipolar spectrum disorders (BSD) [33].

The WURS is designed to retrospectively record symptoms and signs of ADHD in childhood. The version of the scale used in this study contains 25 questions, each rated on a 5-point severity scale. The WURS-25 has been validated by several investigators in different countries and populations [34,35].

The ASRS is the World Health Organization's (WHO) rating scale for adult ADHD designed to measure current ADHD symptoms. It consists of 18 items based on DSM-IV symptoms/criteria for ADHD that are measured on a 5-point scale (0 = never/seldom and 4 = very often), yielding a possible score range from 0-72. The items 1-9 cover the symptoms of inattention; items 10-18 the symptoms of hyperactivity and impulsivity. In this study we used a continuous scoring method [32].

The MDQ is a screening instrument for BSD that has been validated for use in the general population and in psychiatric patient populations [33,36]. The MDQ consists of 15 items. The first 13 questions concern periods of life-time symptoms of mania and hypomania, and the last two ask about co-occurrence of symptoms and ranking of functional impairment caused by the symptoms. A standard MDQ positive score is defined as 7 or more 'yes' on the first 13 items, 'yes' on question 14 (co-occurrence of symptoms) and level '3 or more' on question 15 (moderate to severe impairment).

In addition, self-reported data were collected concerning socio-demographic and clinical factors including educational and occupational levels and co-morbid symptoms and problems. A diagnosis of asthma was defined as answering yes to the following question: "Have you ever had asthma?"

### Statistical analyses

Bivariate associations were analysed using chi-square tests, t-tests for independent samples, and logistic regression analyses. Multivariable associations between ADHD and asthma were examined using a logistic regression model with asthma as the outcome variable, ADHD main predictor, and possible confounders age, gender, education, anxiety/depression and bipolar disorder. All analyses were performed using the Statistical Package for Social Sciences (SPSS) version 15.0.1.

## Results

Clinical and socio-demographic characteristics of ADHD patients and controls are shown in Table 1. In the control group there was a higher proportion of females than in the patient group (59.4% vs. 48.3%), and the mean age was lower (29.6 vs 34.0 years). The level of education was lower in the patient group and the relative number holding an ordinary job was far lower. The proportions of ADHD patients reporting a life-time history of depression and/or anxiety, bipolar disorder and alcohol problems were significantly increased compared to the controls, and scores on all the self report scales for psychiatric symptoms were substantially higher in the ADHD patient group than in the control group. All these differences between ADHD patients and controls were similar for males and females.

**Table 1:** Clinical and socio-demographic characteristics of patients and controls

	Patients	Controls	P
	**N = 594**	**N = 719**	

Age (mean ± SD)	34.0 ± 10.3	29.6 ± 6.5	< 0.001

Gender (% females)	48.3	59.4	< 0.001

Educational level (%)			

Junior high school	26.3	4.0	

Senior high school	50.0	36.4	

College/university	23.7	59.5	< 0.001

Occupational level (%)			

Working	29.1	80.5	< 0.001

Self reported co-morbidity (%)			

Depression/anxiety	68.5	16.1	< 0.001

Bipolar disorder	11.2	1.4	< 0.001

Alcohol problems	23.1	2.1	< 0.001

WURS (score ± SD, range 0-100)	58.6 ± 17.9	17.5 ± 14.0	< 0.001

ASRS (score ± SD, range 0-72)	45.5 ± 12.3	22.9 ± 10.0	< 0.001

MDQ + (%)	48.2	6.3	< 0.001

Asthma(%)	24.4	11.3	< 0.001

A total of 143 of the ADHD patients reported that they had asthma (24.4%), compared with 81 (11.3%) of the controls (OR = 2.53, 1.88-3.41, p < 0.001). Even after controlling for age, gender, education, anxiety/depression, and bipolar disorder in a logistic regression analysis, the OR only decreased slightly, and retained statistical significance (Table 2). The frequency of self-reported asthma was slightly, but not significantly, higher for females in the control group compared with male controls (12.7% vs. 9.2%, OR = 1.43, 0.88-2.34, chi square), but significantly higher among the female ADHD patients compared with the male ADHD patients (30.9% vs. 18.2%, OR = 2.01, 1.36-2.95, p < 0.001, chi square).

**Table 2:** Results from binary logistic regression analysis

	OR	CI	P
Unadjusted	2.54	1.89-3.44	< 0.001

Adjusted for:			

age	2.75	2.02-3.73	< 0.001

+ gender	2.96	2.16-4.03	< 0.001

+ education	2.55	1.80-3.62	< 0.001

+ anxiety/depression	1.91	1.29-2.82	0.001

+ bipolar disorder	1.89	1.28-2.80	0.002

Odds ratios (ORs) with 95% confidence intervals (CIs) for having asthma given a diagnosis of ADHD.

Table 3 shows the same clinical and socio-demographic characteristics as in Table 1 but this time contrasting ADHD patients with and without asthma. Levels of educational and occupational activity were similar for patients with and without asthma. More ADHD patients with asthma reported a life-time history of depression and/or anxiety than ADHD patients without asthma. This difference was still significant after controlling for age and gender using logistic regression analysis (OR = 1.72, 1.11-2.69, p = 0.016). However, whereas 83.6% of male ADHD patients with asthma reported a history of depression and/or anxiety compared with 61.1% in male ADHD patients without asthma, the corresponding figures for female ADHD patients with and without asthma were 72.7% and 71.8% respectively, indicating a potential gender-specific effect, where males with asthma had a relatively higher symptom load. MDQ-score was positive in a larger proportion of ADHD patients with asthma, than in ADHD patients without asthma. The reported levels of ADHD symptoms in childhood (WURS), current ADHD symptoms (ASRS score) and self-reported history of bipolar disorder did not differ between the two groups.

**Table 3:** Clinical and socio-demographic characteristics of patients with and without asthma

	Asthma	Not asthma	P
	**N = 143**	**N = 441**	

Age (mean ± SD)	33.0 ± 10.2	34.5 ± 10.3	NS

Educational level (%)			

Junior high school	30.5	24.8	

Senior high school	51.6	49.6	

College/university	18.0	25.6	NS

Occupational level (%)			

Working	24.8	30.5	NS

Self reported co-morbidity (%)			

Depression/anxiety	76.9	65.8	0.013

Bipolar disorder	13.3	10.6	NS

Alcohol problems	19.7	24.1	NS

WURS (score ± SD, range 0-100)	60.8 ± 17.2	57.9 ± 18.1	NS

ASRS (score ± SD, range 0-72)	45.6 ± 11.8	45.4 ± 12.5	NS

MDQ + (%)	57.8	45.3	0.013

The characteristics of controls with and without asthma are shown in Table 4. Controls with asthma were less likely to be employed compared to controls without asthma. However, when controlling for age and gender in a logistic regression analysis this difference was no longer significant (OR = 0.57, 0.32-1.02). As for the ADHD patients, self-reported depression and/or anxiety was more prevalent in the asthma group, and was still significant when controlling for age and gender (OR = 2.10, 1.22-3.60, p = 0.007). Controls with asthma had more often been diagnosed with bipolar disorders and a larger percentage scored positively on the MDQ-questionnaire than controls without asthma. However, these differences did not reach statistical significance. Interestingly, even among controls, individuals with asthma reported significantly higher levels of current ADHD symptoms (ASRS score) or childhood symptoms (WURS score) than the group without asthma.

**Table 4:** Clinical and socio-demographic characteristics of controls with and without asthma

	Asthma	Not asthma	P
	**N = 81**	**N = 636**	

Age (mean ± SD)	28.5 ± 6.7	29.8 ± 6.5	NS

Educational level (%)			

Junior high school	2.5	4.3	

Senior high school	45.0	35.2	

College/university	52.5	60.6	NS

Occupational level (%)			

Working	70.1	81.9	0.022

Self reported co-morbidity (%)			

Depression/anxiety	27.2	14.5	0.003

Bipolar disorder	2.5	1.1	NS

Alcohol problems	4.9	1.7	NS

WURS (score ± SD, range 0-100)	22.1 ± 15.6	16.9 ± 13.7	0.008

ASRS (score ± SD, range 0-72)	26.0 ± 11.0	22.5 ± 9.8	0.003

MDQ + (%)	10.4	5.8	NS

## Discussion

There are three main findings of the present study. The first is that adult patients with ADHD significantly more often reported a history of asthma, compared to a control population. The second is that controls with self-reported asthma reported more symptoms of ADHD both in childhood and currently, compared to controls without asthma. Finally, asthma in controls and in male ADHD-patients was associated with self-reported depression and/or anxiety.

The ADHD patients in the present study are very impaired as a group, with a low level of education compared to controls, and less than one third being employed in ordinary work. This is in accordance with previous studies showing a low level of occupational functioning in adult patients with persistent ADHD [10,11]. However, we found no indication that ADHD patients with asthma represent a more impaired subgroup of ADHD patients. The level of education, employment status and scores on the ASRS and WURS scales were not significantly different from ADHD patients without asthma. Among the controls there was a difference in employment status for patients with and without asthma, but this difference disappeared when controlling for age and gender.

Females in our control group had a slightly higher prevalence of asthma compared to male controls, but the difference was not statistically significant. Whereas childhood asthma is more common in boys, adult asthma is consistently more prevalent in females [37-39], possibly related to hormonal factors [40,41].

In the previous clinical studies on the relationship between ADHD and asthma where no association was found [23,24], children only were examined. In a study using data from the Norwegian Prescription Database, we showed that patients prescribed drugs to treat ADHD also were prescribed anti-asthma drugs significantly more often than the population at large [27]. In the prescription study we found a weaker relationship between ADHD and asthma in the younger age groups (< 20 years), than in the older age groups (> 20 years), although the associations were significant across all ages. Those findings, together with results from the current study, the study from the National Survey of Children's Health by Blackman et al. [25], and on adults by Secnik, Swensen & Lage [26], offer strong support for the existence of a co-morbidity between ADHD and asthma.

Such a co-morbidity may appear counterintuitive. ADHD and asthma are very different disorders. ADHD is a chronic disorder comprising problems with attention and concentration, combined with behavioural symptoms such as hyperactivity/restlessness and impulsivity [2]. Asthma is a chronic inflammatory disorder of the airways, with episodic worsening, and symptoms related to the respiratory system. However, both ADHD and asthma have similar co-morbid patterns with regard to anxiety- and mood disorders. ADHD exhibits substantial co-morbidity with generalized anxiety disorder, panic disorder, depressive disorders, and bipolar disorder in adults [9]. Asthma is to a similar degree associated with the same anxiety disorders and with bipolar disorder [42,43]. A large number of the ADHD patients in our sample had co-morbid psychiatric disorders [11,29], and male patients with asthma had a particularly high prevalence of depression and/or anxiety. In the control group asthma was also associated with depression and/or anxiety. It is therefore possible that the association between ADHD and asthma is mediated by these other co-morbid disorders.

Much of the current thinking on the pathophysiology and genetics of ADHD has focused on alterations in dopaminergic systems [21], and there is also substantial evidence that dopaminergic mechanisms are involved in mood disorders [44,45]. Dopaminergic systems have not received a similar focus in pathophysiological research on asthma, and are unlikely to explain the cause or pathophysiology of asthma. It is however interesting to note, that dopaminergic receptors are present in sensory nerves in the airways [46], and inhaled dopamine is able to induce bronchodilation during an acute asthma attack [47]. It is therefore possible that changes in dopaminergic systems, or perhaps other signalling mechanisms, could explain part of the associations between ADHD and asthma. Possibly, there could be a subgroup of patients sharing underlying pathophysiological disturbances causing combined symptoms of asthma, ADHD, mood- and anxiety disorders.

Other relevant factors that could help to explain this co-morbidity may be due to risk behaviour associated with ADHD, most notably tobacco smoking. Teenage and adult patients with ADHD have a higher prevalence of smoking in comparison with the general population [48]. It is still a matter of controversy whether active smoking is a cause of asthma, but it is certain to aggravate symptoms among subjects that are prone to asthma before they start smoking [49]. Unfortunately, in the present study, we did not collect information on smoking habits. Another possible etiological factor in relation to tobacco is passive smoking in childhood [50] or prenatal exposure, since children with ADHD presumably have been exposed to this to a larger extent than children without ADHD [51]. Both passive smoking in childhood and prenatal exposure is associated with an increased risk for asthma, both in childhood [52] and among adults [53].

Another possibility is that inflammatory mechanisms may be a common factor for these disorders. Such mechanisms are important in the pathophysiology of asthma [54,55], are may be involved in mood disorders [56,57], and are also postulated to be involved in ADHD [58].

Both ADHD and asthma are associated with obesity. Several studies have indicated a higher than expected prevalence of obesity in ADHD patients [59], and obesity is a risk factor for the development of asthma [60]. In regard to this it is also interesting that obesity leads to a proinflammatory state [60]. Unfortunately we did not collect obesity data in this study (body mass index, waist circumference), so we cannot determine to what extent this may have been a contributing factor.

### Strengths and limitations

Concerning limitations it is evident that we are not studying the whole range of ADHD patients. Not all patients with such problems consult a doctor, and those that are recruited to the present study probably represent a more severely affected group [11]. It is therefore uncertain if the present results are applicable to ADHD patients in general. The ASRS, WURS and MDQ are well-known and widely used auto-questionnaires, and even though they have not been subject to official validations in Norway, validation studies performed in various other populations have found them suitable for use [11,29].

The diagnosis of asthma was made on the basis of "yes-no" answers to a questionnaire. We made no qualification that the diagnosis should have been given by a doctor. In a study from Germany a fairly good agreement was found between answers to such a question compared to a subsequent interview by a physician [42]. Furthermore, we think it is probable that in a country such as Norway, with a strongly subsidised health service, people that think they have asthma will also have consulted a doctor for such a condition. Still, it is possible that we may have underestimated the prevalence of asthma, since Toren et al. [61] found that self-reported asthma was biased in relation to disease severity, that subjects with a mild disease were less prone to report their asthma. On the other hand, the prevalence figure from the control group (11.3%) is in fairly good agreement with epidemiological studies from Norway. In a report based on data from 1998/99 Brogger et al. [37] found a 9.3% prevalence of asthma in Norwegian adults.

## Conclusions

In conclusion, we have shown that adults patients with persistent ADHD have an increased prevalence of asthma compared to controls from the general population, and that controls with asthma report higher levels of both childhood and current ADHD symptoms. This points to a co-morbidity between these two disorders, possibly related to shared risk factors, pathophysiologies and co-morbidities with mood and anxiety disorders. We suggest that future studies should explore underlying pathophysiological mechanisms that may explain the co-occurrence of ADHD and asthma.

## Competing interests

During the past three years JH has been invited as a lecturer by Janssen-Cilag and Novartis, AH by Janssen-Cilag and OBF by Bristol-Meyers Squibb. TME has received an unrestricted grant from AstraZeneca in 2008, and travel support to attend the American Thoracic Society congresses in 2008 and 2011 from GlaxoSmithKline. KJO declare that he has no competing interests.

## Authors' contributions

JH, AH and OBF participated in the design of the study. AH collected and plotted data. OBF performed the statistical analyzes. OBF, JH, TME and KJO drafted the manuscript. All authors contributed to the interpretation of data and revised the manuscript. All authors read and approved the final manuscript.

## Pre-publication history

The pre-publication history for this paper can be accessed here:

http://www.biomedcentral.com/1471-244X/11/128/prepub
